# Estimating the Economic Impact of Climate Change on Cardiovascular Diseases—Evidence from Taiwan

**DOI:** 10.3390/ijerph7124250

**Published:** 2010-12-17

**Authors:** Shu-Yi Liao, Wei-Chun Tseng, Pin-Yu Chen, Chi-Chung Chen, Wei-Min Wu

**Affiliations:** Department of Applied Economics, National Chung-Hsing University, Taichung, Taiwan E-Mails: sliao@nchu.edu.tw (S.Y.L.); tweichun@nchu.edu.tw (W.C.T.); otarublue@hotmail.com (P.Y.C.); pwos.onf@yahoo.com.tw (W.M.W.)

**Keywords:** cardiovascular diseases, climate change, panel model, contingent valuation method

## Abstract

The main purpose of this study was to investigate how climate change affects blood vessel-related heart disease and hypertension and to estimate the associated economic damage. In this paper, both the panel data model and the contingent valuation method (CVM) approaches are applied. The empirical results indicate that the number of death from cardiovascular diseases would be increased by 0.226% as the variation in temperature increases by 1%. More importantly, the number of death from cardiovascular diseases would be increased by 1.2% to 4.1% under alternative IPCC climate change scenarios. The results from the CVM approach show that each person would be willing to pay US$51 to US$97 per year in order to avoid the increase in the mortality rate of cardiovascular diseases caused by climate change.

## 1. Introduction

Recently, much attention has been paid to investigate the potential effects of climate change on infectious diseases. While many countries have adopted various policies to mitigate the effects of global warming on infectious diseases, cold weather, heat waves, and increased seasonal temperature variations have caused substantial numbers of deaths among vulnerable people all over the World. For instance, Keatinge and Donaldson [1] found that coronary and cerebral thrombosis accounted for about half of the cold-related deaths and respiratory diseases were responsible for about half of the rest.

Many of the studies related to the issues of climate change and human health have typically focused on the effects of climate change on epidemiology, physiology and public health. For instance, Saez *et al.* [2], Shen *et al.* [3], Donaldson *et al.* [4], and Rey *et al.* [5] have tried to estimate the relationship between the mortality rate and temperature. They found that the most suitable temperature (*i.e.*, the temperature with the lowest mortality rate) is around 19 degrees Celsius and it may vary in different regions. This implies that extreme low- and high-temperature levels combined with variations in temperature will directly affect human life. In spite of this important observation, however, very few studies have tried to estimate the economic impacts of climate change on human health, especially in relation to cardiovascular diseases. To fill the knowledge gap in this field, this study adopted an econometric approach to quantify the effects of various climatic variables on cardiovascular diseases. Furthermore, the contingent valuation method (CVM) is used to evaluate the economic impacts of climate change on human health. Please note that the cardiovascular diseases considered in this study include Essential Hypertension, Hypertension Heart Disease, Acute Myocardial Infarction, Angina Pectoris, and Acute But Ill-Defined Cerebrovascular Disease.

The remainder of this paper is organized as follows. Section 2 provides background information on the linkages between climate change and cardiovascular diseases. A brief review on the literature related to empirical and theoretical aspects of the climatic factors and cardiovascular diseases is included in this section. Section 3 presents the methodology and the panel model regression approach applied to estimate the relationship between the climatic conditions and the number of deaths resulting from cardiovascular diseases. Section 4 estimates the economic impacts of climate change on cardiovascular diseases by using a non-market valuation approach. Finally, our conclusions and policy suggestions are presented in Section 5.

## 2. Background to Climate Conditions and Cardiovascular Diseases

In general, a healthy individual will be able to effectively cope with thermal stress through the efficient regulation of heat. Changes in blood pressure, blood viscosity, and heart rate associated with physiological adjustments to cold and warmth, however, may explain the temperature-induced increase in mortality due to diseases of the cardiovascular system [6]. Therefore, the risk of death will be substantially increased if the changes of temperature exceed the limits that the cardiovascular system can withstand. Such increases in mortality from cardiovascular diseases may result from changes in blood pressure, blood viscosity, and heart rate associated with physiological adjustments to cold and warmth [6].

Such a relationship between human health and climate change has been investigated by many studies. For instance, Alfésio *et al.* [7] found that temperature, latitude, and the mortality rate are highly correlated in 11 Eastern areas in the U.S. Donaldson *et al.* [8] appraised the cardiovascular disease mortality rate during the winter season in Russia, and found that the mortality rate increases when the average daily temperature is below zero degrees Celsius. Basu and Samet [9] used a time series approach to estimate how the mortality rate is affected by heat waves, found that the mortality rate increases due to the increase in the duration and intensity of heat waves. Alfésio *et al.* [7] found that the mortality rate from cardiovascular disease increases when there are extremely high or low temperatures. However, Basu and Samet [9] also pointed out that urban pollution is a principal factor explaining deaths based on the relationship between human health and climate change. Ebi *et al.* [10] found that the variations in climate due to the ENSO phenomenon may help explain the increase in cardiovascular disease in hospital populations. All these studies have shown that human health is significantly affected by the level of and variation in climate-related variables. This implies that the level and variation in temperature are the two major factors affecting the mortality related to cardiovascular diseases.

In spite of the voluminous research concerned with the relationship between mortality and climatic conditions, however, very few studies have tried to empirically investigate the effects of changes in climatic conditions on mortality. Because empirical estimations can provide valuable information and policy implications in the fields of public health, medicine, health insurance and the environment, it is very import to further explore and quantify the relationship between mortality and climatic conditions in the context of cardiovascular diseases. In this paper, we conducted a case study on Taiwan to demonstrate how the effects of changes in climate conditions on the mortality in relation to cardiovascular diseases can be estimated.

**Figure 1:**
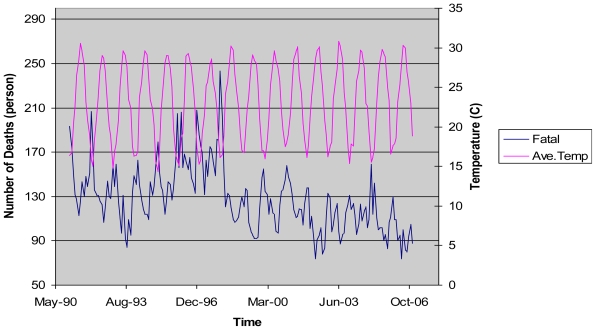
The Average Temperature and the Number of Deaths from Cardiovascular Diseases from Years 1991 to 2006.

**Figure 2:**
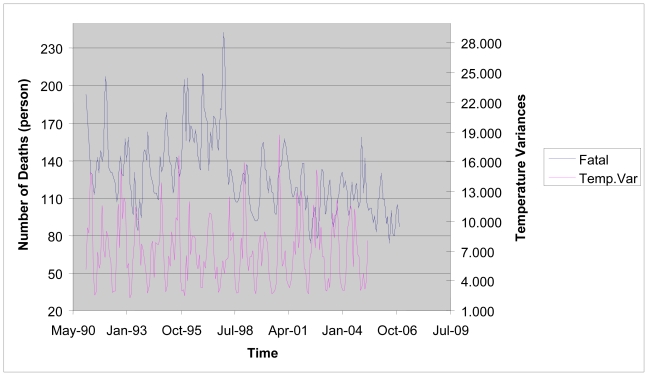
The Temperature Variation and the Number of Deaths from Cardiovascular Diseases from Years 1991 to 2006.

**Figure 3:**
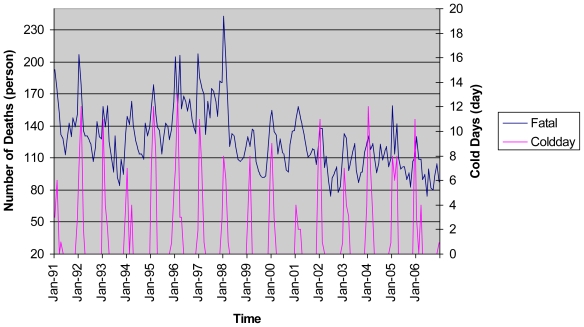
The Cold Days and the Number of Deaths from Cardiovascular Diseases from Years 1991 to 2006.

The temperature in Taiwan has gradually increased in a manner consistent with global warming. The annual average temperature during the 1960s was about 22.8 degrees Celsius, but it has increased to 24.0 degrees Celsius during the first decade of the 21st century. The historical trends of the average temperature, the temperature variation, and the number of cold days along with the number of deaths from cardiovascular diseases in Taiwan during the period from 1991 to 2006 are shown in Figures 1 to 3. Figure 1 reveals that both the average temperature and the number of deaths from cardiovascular diseases exhibit seasonal cycles. It also shows that the number of deaths from cardiovascular diseases tends to increase (decrease) as the average temperature decreases (increases). This implies that there may exist a negative relationship between the average temperature and the number of deaths from cardiovascular diseases. As presented in Figure 2, we observe that higher numbers of deaths from cardiovascular diseases are generally associated with higher temperature variations during winter months. Therefore, increases in the temperature variation could potentially cause more people died from cardiovascular diseases. Besides, we find that the number of cold days could also affect the number of deaths from cardiovascular diseases as shown in Figure 3. In Taiwan, the number of cold days in a month is defined as the number of days with the lowest temperature less than 12 degrees Celsius. As more cold days occur in a month, people with cardiovascular diseases will have a higher risk of mortality. Although Figures 1 to 3 have provided strong evidence of a significant relationship between climatic conditions and the number of deaths from cardiovascular diseases, the magnitude of such effects needs to be further investigated to provide meaningful data to clarify the policy implications for the government.

## 3. Estimating the Effects of Climate Change on Cardiovascular Diseases

### 3.1. Model Equation Specifications

To estimate the effects of climate change on the mortality of cardiovascular diseases in Taiwan, a two-step estimation approach is applied. Such an estimation procedure is similar to that in the study by Tseng *et al.* [11]. As the first step, the relationship between the number of deaths from cardiovascular diseases and various climatic conditions needs to be established and estimated. The estimation outcomes with the combinations of alternative climate change scenarios are adopted in the second step. To determine the effects on mortality of thermal stress in relation to cardiovascular diseases due to changes in climatic conditions, both the possible climatic conditions and socio-economic factors need to be specified. Based on the review of the literature in the previous section, the possible climate conditions include the average temperature, the lowest temperature, the temperature variation, the number of cold days, precipitation, humidity, and seasons.

The mortality from cardiovascular disease function takes the form of Equation (1):

(1) $$\begin{array}{l} {Death}_{it}=F({ATTEMP}_{it},{MINTEMP}_{it},{RAIN}_{it},{WET}_{it},{COLDDAY}_{it},{VARTEMP}_{it},\\ {SEASON}_{it},{SEASON}_{it}\times {VARTEMP}_{it},{SEASON}_{it}\times {MINTEMP}_{it},\\ {POPUL}_{it},HEALTHIN)+{ɛ}_{it} \end{array}$$

where *Death_it_* is the monthly number of deaths from cardiovascular disease in region *i* in period *t*, *ATTEMP_it_* is the monthly average temperature in region *i* in period *t*, *MINTEMP_it_* is the monthly mean of the daily lowest temperature in region *i* in period *t*, *RAIN_it_* is the monthly precipitation in region *i* in period *t*, *WET_it_* is the relative humidity in region i in period t, *COLDDAY_it_* is the monthly cold days with the lowest daily temperature below 12 degrees Celsius in region i in period t, *VARTEMP_it_* is the monthly variation in temperature in region i in period t and is calculated based on the sampling variance using daily average temperature. *SEASON_it_* is a seasonal dummy variable, which equals 1 for the winter season and 0 otherwise, *POPUL_it_* is the monthly population in region i in period t, *HEALTHIN* is a dummy variable for health insurance, which equals 1 when national health insurance is implemented and 0 otherwise.

### 3.2. Datasets

Two different datasets are compiled for the estimation of Equation (1). The first dataset include the monthly number of deaths from cardiovascular disease for each region in Taiwan. The second dataset include all climatic condition variables in Equation (1). The sources of the first and second dataset are retrieved from the Department of Health, Executive Yuan in Taiwan, and the International Research Institute for Climate and Society (IRI), respectively. Data sets for 13 regions based on the administration system in Taiwan were collected. The combined dataset is consisted of monthly data from January 1971 to December 2006 for each region. Therefore, there are 432 monthly observation data for 13 regions included in our balanced panel model. The basic descriptive statistics for each variable are presented in Table 1.

Table 1 shows that the monthly number of deaths from cardiovascular diseases in a region ranges from 0 to 271 persons with an average of about 55 persons. This implies that the level of the mortality rate varies significantly between different months. Levels and variations of climate conditions as well as seasons may contribute such an unsteady phenomenon. The average monthly temperature is about 23.38 degrees Celsius, which seems to be favorable for human beings, however, the minimum average temperature occasionally falls down to 13 degrees Celsius and the maximum at times reaches up to 31 degrees Celsius. This significant difference in terms of average monthly temperature may affect the numbers of people in Taiwan suffering from cardiovascular diseases. The mean and variance of the monthly variation in temperature (VARTEMP) are 4.2% and 11.18%, respectively, while the maximum sometimes reaches up to 21.22%.

The statistics related to the precipitation and humidity characteristics of Taiwan are displayed in the fifth and sixth rows of Table 1, respectively. In general, Taiwan has more rainfall and higher humidity than the world averages. The statistical characteristics for the two extreme event variables including the number of cold days (*i.e.*, COLDDAY) and the lowest monthly temperature (*i.e.*, MINTEMP) are shown in the seventh and eighth rows of Table 1, respectively. In general, people with cardiovascular diseases have a higher risk of mortality in cold days compared to non-cold days. The mean of the COLDDAY variable is about 3.68 days, while the maximum could reach as many as 30 days. This implies that the risk of cardiovascular diseases increases as the number of cold days increases. The other extreme event is referred to as MINTEMP which measures the average of the lowest daily temperature. The mean of the MINTEMP variable is about 20.44 degrees Celsius, which is lower than the mean of the average monthly temperature.

### 3.3. Estimation Results

Our dataset is a panel dataset with time series data for each variable by region. We applied both the fixed effects and the random effects models to estimate Equation (1). The estimation outcomes are shown in Table 2. Based on the Hasuman test, the fixed effects model is rejected which indicates that the random effects model is an appropriate model. Several important findings can be drawn from the estimation outcomes. The first finding is that the number of deaths from cardiovascular diseases will decrease if the average monthly temperature (ATTEMP) goes up, which is consistent with the findings of Keatinge and Donaldson [1]. They concluded that the global warming will reduce the number of deaths from heart disease. The estimated parameter indicates that the percentage of the number of deaths from cardiovascular diseases will decrease by 0.231% if the average temperature increases by 1%. However, the estimated cross effect of the seasonal dummy variable and the average temperature shows that the effect of a 1% decrease of average monthly temperature on the mortality of cardiovascular disease in the winter season will be 0.132% higher than that in the other seasons. The positive sign of seasonal dummy variable indicates that the mortality of cardiovascular disease is more sensitive during winter seasons.

Secondly, the empirical results indicate that the percentage of the number of deaths from cardiovascular diseases will increase by 0.226% if the variation in monthly temperature increases by 1%. This implies that the variation in temperature (VARTEMP) has a significant positive effect on the number of deaths from cardiovascular diseases. The effect of precipitation is significantly positive while the effect of the daily lowest temperature (MINTEMP) is significantly negative which implies that the lower temperature will increase the mortality of cardiovascular disease.

Thirdly, for the extreme weather events, we found that the number of deaths from cardiovascular diseases is significantly affected by the number of cold days (COLDDAY). The percentage of the number of deaths from cardiovascular diseases will increase by 0.277% if the number of cold days increases by 1%. The empirical results also indicate that bigger population will result in higher mortality and a good health care system can reduce mortality.

According to climate change scenarios on temperature, the estimated parameters shown in Table 2 can be used to calculate the effects of climate change on the mortality of cardiovascular diseases in Taiwan. Our empirical results indicate that the effect of the average monthly temperature on the number of deaths from cardiovascular diseases is more profound than those of the other climate variables. As a result, the estimated parameter can be used to calculate the effects of climate change on the mortality of cardiovascular diseases for alternative climate change scenarios on temperature. The climate change scenarios are retrieved directly from IPCC’s 2007 Report [13] which includes scenarios B1, B2, A1B, and A2 at year 2100. Among the four scenarios, B1 and B2 are more optimistic scenarios while A1B and A2 are more pessimistic scenarios. The best estimated temperature rise is 1.8 °C with a likely range of 1.1 to 2.9 °C for scenario B1, while the best estimated temperature rise is 2.4 °C with a likely range of 1.4 to 3.8 °C for scenario B2. For the two pessimistic scenarios A1B and A2, the best estimated temperature rise are 2.8 °C (ranging from 1.7 to 4.4 °C) and 3.4 °C (ranging form 2.0 to 5.4 °C), respectively. Based on IPCC’s estimation of global climate sensitivity and a case study on the projected climate change in Taiwan by Hsu and Chen [12], the temperature variation increase in Taiwan due to climate change was simulated to be within the range of 5% to 16%. The estimated effect of temperature variations on the number of deaths from cardiovascular diseases is 0.235% for the winter season and 0.448% for the other seasons when the temperature variation increases by 1%. Therefore, the combination of these two numbers could be used to estimate the possible percentage increase in the number of deaths from cardiovascular diseases due to climate change. In this study, we adopted the lowest value (1.2%) and the middle value (4.1%) of the estimated range to evaluate the economic impacts of climate change on cardiovascular diseases.

## 4. Evaluating the Damage from Cardiovascular Diseases due to Climate Change

The previous section has revealed the severity of the impact of climate change on the mortality of cardiovascular diseases. This implies that an economic assessment of climate change induced health threat to human beings is also very important in view of the decision-making involved in public health investments to mitigate the damage caused by such diseases. To estimate the economic impacts of climate change on cardiovascular diseases, the contingent valuation method (CVM) approach is applied in this paper. The survey-based contingent valuation method is used to look into the willingness to pay (WTP) of respondents in a particular region or country. For public health agencies, the information regarding WTP for a reduced exposure to the health threat is often needed to acquire funding to cover the administrative costs of surveillance and reporting as well as the costs of the scientific development in disease control.

### 4.1. Modeling Design for the CVM

The CVM was originally proposed by Davis [14] to evaluate the value of non-market or environmental goods. Since then, this method has been widely applied to cases of non-market goods including on ecological, environment, recreation, and health areas. The CVM allows one to elicit from individuals a definite indication of their preferences when it is difficult to observe this behavior in an existing market. Furthermore, the estimated results could lead to compensated measurements of welfare, which are expressed in monetary values. The CVM employs specific surveys designed on the basis of the need to induce respondents to reveal their WTP for the object being evaluated. Its principle consists of confronting individuals with a hypothetical market in which non-market goods are traded. Then, the respondents are asked to disclose their preferences for the goods traded by means of a bidding process (Tseng *et al.* [11]).

The single-bounded dichotomous choice (SBDC) model developed by Bishop and Heberlein [15] has been widely used to estimate the economic value of non-market goods. Based on the SBDC model, respondents are asked to bid with the answer of “yes” or “no” to express their willingness in a questionnaire. Therefore, the SBDC model is suitable for the estimation of the WTP to reduce the exposure to health threats. In this study, we also use SBDC in the questionnaire to investigate the respondents’ WTP.

To determine the bids in the questionnaire, a pretest was conducted over a period of five days from May 2 to May 5, 2008. The respondents were asked to fill out an open-ended questionnaire, which revealed information concerning the distribution of their WTP. A total of 30 people were sampled in this pretest. According to Alberini [16], we ranked the WTP of these valid samples and selected 13 groups to be the designated bids as shown in Table 3 in the formal survey.

Below is a commonly used grouped data model for the CVM that we applied in this study:

(2) $$\begin{array}{c} y^{*}=\beta^{\prime }x+ɛ,\;\;\;ɛ∼N[0,\sigma^{2}]\\ y=k\;\;\;\text{if }A_{k-1}<y^{*}\leq A_{k},\;\text{k}=1,2,\ldots ,\text{K},\;A_{0}=-\infty ,A_{K}=\infty , \end{array}$$

where y* is the latent (actual) WTP but is not observable to the researchers. The researchers only know which category the respondent chose. *β* is a vector of parameters, *x* is a matrix of explanatory variables, and *ɛ* is the error term with normal distribution. In this model, please note that it is not necessary to normalized *σ* into 1 but we chose to do so to better explain the estimation results.

The probability of people being affected by cardiovascular diseases was expected to increase due to increased variations in temperature and precipitation resulting from the climate change. Therefore, in the design of our questionnaire, we assumed that the government will establish an authority to implement some surveillance measures and actions to reduce the possibility of cardiovascular diseases. For example, the authority could provide accurate information on variations in temperature, educate people on how to prevent these diseases, and promote regular health checks. The funding source involves levying an extra tax on the general population each year.

Based on the calculations in the previous section, the probability of death from cardiovascular diseases would increase by a range of 1.2% to 4.1% due to the future climate change in Taiwan. Therefore, the WTP for the lowest and the highest cases will be investigated in this study. The survey was conducted over a period of three weeks from May 7 to May 25, 2008. The questionnaire consists of four parts, including the knowledge regarding climate change, awareness of cardiovascular diseases, the major WTP questions and bids, and socio-economic background. Each respondent was asked to fill out a questionnaire which elicited information concerning his/her WTP to reduce the probability of death from cardiovascular diseases affected by climate change. We obtained a total of 524 samples. After eliminating the protest observations, 510 observations were left. Among the 510 samples, males and females accounted for about 42% and 58% of the total, respectively. As for income, most of the respondents’ monthly income range from NT$61,000 to 70,000.

More than 98% of the respondents were aware of the climate change and more than 94.5% were quite concerned about this issue. They attached great importance to global warming and thought the government should implement some policy measures to reduce greenhouse gas emissions. Almost all respondents knew about the cardiovascular diseases and were also concerned about the impact of variations in temperature on these diseases. Among the 510 samples, 12.7% of correspondents were suffering from cardiovascular diseases at the time of the survey and 91% of correspondents had family members suffering from these diseases. However, only 35% of correspondents had a regular health check, although at least 94% of them had adopted some prevention strategies such as preparing suitable clothing and maintaining a balanced diet to prevent such diseases.

The mortality rate of cardiovascular diseases could be affected by several major factors such as diet, weather, and medical facilities. Since we focus on the possible increase in the mortality rate of cardiovascular diseases caused by the climate change, thus in the questionnaire we inform the respondent of the risk associated with climate. Alternatively, under a more general questionnaire, it would be simpler to just ask for WTP for reductions in risk without specifying the origin of the risk source.

To estimate the economic impacts of cardiovascular diseases due to climate change, the CVM is applied here. Since many factors including the cognition of the objects, bids, and backgrounds of respondents will affect the respondent’s WTP, several important explanatory variables were selected as shown in Table 4. Table 4 provides the definitions, expected signs, and the summary statistics for the selected variables.

When the questionnaire is in the payment card format in order to select the WTP under various mortality risks, the WTP range should be transformed into grouped data format [Cameron and Huppert 17]. Therefore, we also transformed the dependent variable into the grouped data format to estimate the WTP function.

Suppose the respondent’s WTP is a function of the explanatory variables shown in Table 4, we can express the empirical model as Equation (3) below:

(3) $$\begin{array}{l} {WTP}_{ij}^{*}=\beta_{0}+\beta_{1j}{WARM}_{ij}+\beta_{2j}{VARTEMP}_{ij}+\beta_{3j}{ENVIRONMENT}_{ij}+\beta_{4j}{KNOW}_{ij}\\ +\beta_{5j}{EFFECTED}_{ij}+\beta_{6j}{CHECK}_{ij}+\beta_{7j}{PREVENT}_{ij}+\beta_{8j}{RVALUE}_{ij}+\beta_{9j}{SEX}_{ij}\\ +\beta_{10j}{AGE}_{ij}+\beta_{11j}{EDU}_{ij}+\beta_{12j}{MAR}_{ij}+\beta_{13j}{FAMILY}_{ij}+\beta_{14j}{INCOME}_{ij}+\beta_{15j}{INSU}_{ij}+{ɛ}_{ij} \end{array}$$

where *β_0_* is the intercept, and *β_1j_*, *β_2j_*, …, *β_15j_* are the coefficients of the explanatory variables. WTP_ij_* is the unobservable actual WTP of the *i*th respondent in the *j* case and *j* =1, 2 stands for cases 1 and 2. Because the researchers can only observe the categories that each respondent chose, we followed the suggestions by Cameron and Huppert [17] and transformed the data into grouped data format. In this study, we used the information from the second column of Table 3 to define Case 1 as

(4) $$\begin{array}{c} y=1\;\;\;\text{if }\;\;{WTP}_{i1}^{*}\leq 250\\ 2\;\;\;\text{if }\;\;250<{WTP}_{i1}^{*}\leq 500\\ 3\;\;\;\text{if }\;\;500<{WTP}_{i1}^{*}\leq 750\\ 13\;\;\;\text{if }\;\;3000\leq {WTP}_{i1}^{*} \end{array}$$

Similarly, we used the data from the thrid column of Table 3 to define Case 2. The empirical model is estimated using the grouped data model in the econometric software LIMDEP 8.0. The likelihood ratio test (LRT) and the correct predicted percentage were used to measure the goodness-of-fit of the empirical model. The formula for the likelihood ratio is:

(5) $$LR=-2\text{ln}\;(L_{w}/L_{\Omega })=-2(\text{ln}\;L_{w}-\text{ln}\;L_{\Omega })$$

In equation (5)*L_w_* is a restricted maximum likelihood value while *L_Ω_* is an unrestricted maximum likelihood value. The likelihood ratio yields a chi-square distribution. A high LR value means that the model exhibits significant explanatory ability. The correct predicted percentage is calculated based on the number of correct predicted samples divided by the total sample size. A high correct predicted percentage implies a high validity of the model.

The empirical results are presented in Table 5. In terms of the signs of the estimated parameters, the results confirm what we expected in Table 4. The estimates for cases 1 and 2 are consistent with each other in terms of the signs and the statistical significance of the parameters. The results reveal that income appears to have an important influence on WTP. Besides, the results indicate many variables have significant positive effects on WTP including the respondents’ sense of the climate change (WARM), the variation in temperature (VARTEMP), the respondents’ sense with respect to climate change on the environment (ENVIRONMENT), whether the respondent already suffers from cardiovascular diseases (EFFECTED), risk behaviors (RVALUE), and age (AGE). On the contrary, only the health check (CHECK) and prevention behavior (PREVENT) have significant negative effects on WTP. The results also suggest that sex, marriage, education and the number of family members are not significant variables. It is worth noting that respondents with health insurance have relatively higher WTP than those without health insurance. Knowledge of the mortality from cardiovascular diseases has significant positive effects in Case 2, but it does not have significant effects in Case 1.

Based on the empirical results, we can calculate the public’s WTP for the two cases by using the function of *WTP* *_mean, j_* *=* β̂′ *x̄ _j_* where β̂ *_j_* is a vector of estimated parameters and *x̄_j_* is a vector of means of explanatory variables. People would be willing to pay NT$1,685 (*i.e.*, US$ 51) and NT$3,212 (*i.e.*, US$ 97.3) per year to reduce the probability of death from cardiovascular diseases in Case 1 and Case 2, respectively. There are two meanings attached to these figures. The first interpretation is that the economic damage from cardiovascular diseases due to climate change ranges from NT$1,685 to NT$3,212 per year per person. Therefore, for the entire society, the total economic damage ranges from NT$29.16 to NT$55.59 billion (or US$0.88 to US$1.68 billion) per year. The second interpretation is that people in Taiwan would be willing to pay these amounts of taxes to the government for them to take action to reduce such health risks.

The above estimated values could be justified by comparing them with other WTP estimates for other human diseases. For instance, Tseng *et al.* [11] estimated the effects of climate change on dengue fever, an infectious disease in Taiwan, and found that the respondents’ WTP to avoid the increasing probability of dengue fever by climate change ranged from US$0.52 to US$3.67 billion. As another extreme example, Liu *et al.* [18] estimated the WTP to reduce the risk of infection and death from severe acute respiratory syndrome (SARS) in Taiwan and found that the values per statistical life ranged from 3 to 12 million US dollars. Their WTP estimates are higher than those of Tseng *et al.* [11] and this study because of the higher mortality rate associated with SARS.

Comparing with other diseases such as the food-borne diseases Campylobacteriosis or Salmonellosis, Goldberg and Roosen [19] applied CVM and found that an individual’s WTP ranged from US$2.98 for a risk reduction of 40% for Campylobacteriosis or Salmonellosis to US$5.88 for a risk reduction of 80% for Salmonellosis and a risk reduction of 40% for Campylobacteriosis. Sauerborn *et al.* [20] found that people would be willing to pay US$2.58 to US$3.78 for a vaccine against maternal malaria in Burkina Faso. A couple of studies also provide WTP estimates for noninfectious diseases. Kleinman *et al.* [21] found that respondents would be willing to pay up to US$181.66 to obtain a complete relief in a short period of time without the side effects of Gastroesophageal Reflux, while Johnson *et al.* [22] found that respondents would be willing to pay $1,510 over three years to participate in a program to prevent the onset of diabetes. All of the studies mentioned above indicate that the respondents’ WTP depends on the types of diseases, the mortality rate, as well as the country surveyed. We found our estimation values to be within the middle range of these estimates.

Although hot days could also affect the mortality of cardiovascular diseases [23,24], however, cardiovascular diseases are generally more sensitive to cold days than to hot days. For example, Braga *et al.* [25] analyzed data from USA cities and found that the effect of cold days on the cardiovascular diseases is five times of that caused by hot days. In addition, Figure 1 also shows that the mortality rate is relatively higher when the temperature is relatively lower in Taiwan. On the contrary, the mortality rate is relatively lower when the temperature is relatively higher. Thus we did not include hot days in our regression model and may be included in future studies.

## 5. Concluding Comments

While the relationship between mortality and temperature has been recognized in several different regions by many studies, little attention has been paid to estimate the effects of climatic conditions on the mortality of cardiovascular diseases. In this paper, a case study on Taiwan is performed as an example to estimate how climatic conditions affect the number of deaths from cardiovascular diseases and how much the damage will be when climate change is incorporated into the model.

The relationship between climate conditions and the number of deaths from cardiovascular diseases is assessed using a panel data regression model based on monthly climate and cardiovascular disease data during the period from 1971 to 2006 in Taiwan. The empirical results show that the mortality rate of cardiovascular diseases would increase by 0.226% as the temperature variation increases by 1%. We found that the mortality rate of cardiovascular diseases would increase by a range of 1.2% to 4.1% with respect to alternative climate change scenarios. The CVM is also applied to estimate the economic impacts of climate change on cardiovascular diseases. The respondent’s WTP is investigated in the survey using the payment card approach. The results show that each person would be willing to pay US$51 to US$97 per year (or a total of US$880 million to US$1,680 million for the whole public) in order to avoid 1.2% to 4.1% increase in the mortality rate of cardiovascular diseases caused by climate change.

Our estimates of the economic impacts of climate change on the mortality of cardiovascular diseases provide important policy implications for the government. The estimated WTP provides a valuable basis for the government to measure the cost of medical treatments for cardiovascular diseases, which may increase rapidly due to climate change. Therefore, the government could use people’s WTP to reduce the mortality of cardiovascular diseases to treat patients with cardiovascular diseases or to prevent the onset of such diseases.

## Figures and Tables

**Table 1:** Descriptive Statistics.

	
	Unit	Mean	Variance	Maximum	Minimum
***Death***	Person	55.43	1,732.00	271.00	0.00
***ATTEMP***	Celsius	23.38	19.32	31.00	13.00
***VARTEMP***	%	4.20	11.18	21.22	0.13
***RAIN***	Mm	173.44	37,419.35	1,860.00	0.00
***WET***	%	87.00	387.07	121.50	39.60
***COLDDAY***	Day	3.68	44.08	30.00	0.00
***MINTEMP***	Celsius	20.44	19.23	27.90	8.80
***POPUL***	Person	1162,946	890,933,617,891	3,900,163	88,855

**Table 2:** The Estimation Results for the Cardiovascular Disease Equation.

Variable	Parameter estimates	
	Fixed effects Model	Random effects Model
*CONSTANT*	4.656*** (0.606)	3.920*** (0.950)
log *ATTEMP*	−0.048** (0.025)	−0.231*** (0.032)
log *VARTEMP*	0.031 (0.037)	0.226*** (0.051)
log *MINTEMP*	0.099 (0.149)	−1.516*** (0.206)
log *RAIN*	0.000 (0.003)	0.053*** (0.004)
log*WET*	−0.282*** (0.083)	−0.749*** (0.106)
log *COLDDAY*	0.142 (0.092)	0.277* (0.157)
log *POP*	0.308*** (0.010)	0.575*** (0.005)
*SEASON*	0.568** (0.248)	0.644** (0.294)
*HealthIC*	−0.172*** (0.013)	−0.238*** (0.014)
log ATTTEMP* log *VARTEMP*	0.011 (0.011)	0.071 (0.016)
SEASON* log *ATTEMP*	−0.099** (0.043)	−0.132*** (0.051)
SEASON* log *VARTEMP*	−0.040 (0.086)	−0.213* (0.123)

**Adjusted R^2^**	0.906	0.557

**Hausman Test**		10.389

Note: The numbers in the parentheses are the standard deviations while

* denotes statistical significance at the 10% level,

** represents the 5% significance level, and

*** represents the 1% significance level.

**Table 3:** Bids of WTP Using the Payment Card Format.

Bids	1.2% Reduction Case	4.1% Reduction Case

1	Under NT$ 250	Under NT$ 500
2	NT$ 251~500	NT$ 501~1,000
3	NT$ 501~750	NT$ 1,001~1,500
4	NT$ 751~1,000	NT$ 1,501~2,000
5	NT$ 1,001~1,250	NT$ 2,001~2,500
6	NT$ 1,251~1,500	NT$ 2,501~3,000
7	NT$ 1,501~1,750	NT$ 3,001~3,500
8	NT$ 1,751~2,000	NT$ 3,501~4,000
9	NT$ 2,001~2,250	NT$ 4,001~4,500
10	NT$ 2,251~2,500	NT$ 4,501~5,000
11	NT$ 2,501~2,750	NT$ 5,001~5,500
12	NT$ 2,751~3,000	NT$ 5,501~6,000
13	Above NT$ 3,000	Above NT$ 6,000

**Table 4:** The Definition, Expected Sign, and Descriptive Statistics for Each Variable.

Variables	Definitions	Expected Sign	Mean	Standard Deviation
WARM	The respondent’s sense on climate change (1: know climate change; 0: otherwise)	+	0.988	0.108
VARTEMP	The respondent’s sense on temperature variation(1: has sense; 0: otherwise)	+	0.903	0.294
ENVIRONMENT	The respondent’s sense with respect to climate change on environment (level is ranked from 1 to 5, the higher level represents a higher sense)	+	4.382	0.636
KNOW	The respondent’s knowledge of mortality from cardiovascular diseases (1: yes; 0: no)	+	0.945	0.228
EFFECTED	Whether the respondent has cardiovascular diseases (1: yes; 0: no)	+	0.127	0.333
CHECK	The respondent’s regular health checking status (1:yes; 0: no)	−	0.349	0.477
PREVENT	The respondent’s prevention of cardiovascular diseases (1:yes; 0: no)	−	0.994	0.076
RVALUE	The respondent’s risk sense of cardiovascular diseases (ranked from 1 to 5, the higher the value, the more risk averse)	+	4.451	0.513
SEX	The respondent’s sex (1: male; 0: female)	+/−	0.417	0.493
AGE	The respondent’s age	+/−	36.0	9.7
EDU	The respondent’s educational level (1: university and above; 0: below university level)	+	4.06	0.55
MAR	The respondent’s marriage situation	+/−	0.720	0.449
FAMILY	The number of the respondent’s family members	+/−	4.692	1.071
INCOME	The respondent’s income per month	+	26,000	16,700
INSU	The respondent’s insurance status (1: yes; 0: no)	+/−	0.998	0.044

**Table 5:** Estimation Results of WTP.

Variables	Case 1 (1.2% )	Case 2 (4.1%)
Constant	1079.3*** (318.0426)	1094.49 ** (534.6716)
WARM	341.501* (202.3433)	745.374* (397.9487)
VARTEMP	197.229*** (73.5268)	773.109** (387.8304)
ENVIRONMENT	89.3973*** (25.0579)	195.727*** (69.9267)
KNOW	264.064 (164.4316)	558.456* (324.4064)
EFFECTED	165.492*** (49.5750)	314.032*** (94.5543)
CHECK	−34.7412** (14.3495)	−121.917*** (45.4615)
PREVENT	−664.102** (333.4012)	−1032.26** (469.6723)
RVALUE	181.252*** (68.5267)	389.731*** (135.1697)
SEX	18.5622 (73.6352)	70.6269 (145.2275)
AGE	85.9913*** (31.8430)	187.333** (94.4909)
EDU	−81.3023 (66.4489)	−175.813 (130.9028)
MAR	12.3434 (83.9498)	−38.7384 (165.5515)
FAMILY	−18.1825 (33.6511)	−36.6846 (66.4106)
INCOME	9.93199** (5.0073)	48.9428** (24.6238)
INSU	−514.298* (309.5905)	−893.778** (380.6193)
*WTP* Mean	1685.26	3212.46
*WTP* Median	1697.16	3209.57
Log likelihood	−1170.518	−1167.725
LRT (Chi-squared)	44	44.142
Sample size	510	510

Notes: (1) The numbers in the parentheses are the standard deviations while

* denotes statistical significance at the 10% level,

** represents the 5% significance level, and

*** represents the 1% significance level. (2) *χ*^2^ (0.01,10) = 23.29.
